# From stress to success: using physiological data to predict cardiopulmonary resuscitation simulation performance

**DOI:** 10.3389/fpsyg.2026.1659195

**Published:** 2026-03-05

**Authors:** Luca Queirolo, Giulia Mormando, Anna Vittadello, Giulia Cason, Barbara Maino, Tommaso Pettenuzzo, Nicolò Sella, Francesco Zarantonello, Annalisa Boscolo, Gastone Zanette, Paolo Navalesi

**Affiliations:** 1 Department of Neuroscience, University of Padua, Padua, Italy; 2 Istituto Franco Granone-Centro Italiano di Ipnosi Clinico Sperimentale, Turin, Italy; 3 Department of Philosophy, Sociology, Education and Applied Psychology, University of Padua, Padua, Italy; 4 Department of Medicine (DIMED), Padua, Italy; 5 Institute of Anesthesia and Intensive Care, University Hospital of Padua, Padua, Italy

**Keywords:** cardiopulmonary resuscitation (CPR), electro dermal activity (EDA), emergency medicine, heart rate variability (HRV), performance, stress

## Abstract

**Background and aim:**

Managing stress is critical in emergency medicine, where cardiopulmonary resuscitation (CPR) rely on team dynamics. Although subjective and physiological markers assess stress, few studies have examined their combined effects during CPR simulations. The influence of team role (leader vs. member) and whether physiological data can predict performance also remain underexplored. This study addresses these gaps.

**Methods:**

Thirty emergency residents attending the School of Emergency Medicine of the University of Padua (Italy) were recruited with previous certification in Advance Cardiac Life Support (ACLS) and randomly paired, each assigned to one of two roles: team leader (TL) or team member (TM). Randomization also considered baseline stress level (PSS-10). Each pair was then assigned to cardiac arrest with a shockable or non-shockable rhythm, including 2 min of uninterrupted chest compressions, following American Heart Association (AHA) guidelines. The data collected included CPR performance metrics (compression depth, rate, recoil). Physiological data were collected before, during, and after CPR using Empatica E4 and eSense, Heart rate (HR), Heart Rate Variability (HRV), Electrodermal Activity (EDA), and Skin conductance response (SCR).

**Results:**

Participants reported moderate baseline stress (PSS-10, VAS stress/anxiety). Baseline physiological measures were within normative ranges. ANOVA revealed a significant effect of group condition for HRV (*p* < 0.05); HR significantly increased from baseline to CPR (*p* < 0.001) and decreased post-CPR (*p* < 0.001). EDA increased from baseline to both CPR and post-CPR (*p* < 0.001). No significant differences were found between team roles at exception for HRV. Binomial logistic regression models using sympathetic data did predict CPR performance (TM EDA Pre, TL EDA Pre and TL SCR pre simulation, *R*^2^ = 0.39, AIC = 19.804, *p* < 0.05, accuracy = 0.8667). Furthermore, a nonlinear regression using HRV-derived SD1 predicted performance (*R*^2^ = 0.56; coefficient *a*, *p* < 0.01; coefficient *b*, *p* < 0.01).

**Conclusion:**

This study shows that simulated CPR scenarios trigger psychophysiological stress responses. Increased HRV, HR, and EDA indicate a challenge-type reaction, despite stable subjective ratings across team roles, suggesting a shared load, with TL sympathetic activation as a possible mediator of global team activation. Notably, a nonlinear link between SD1 and performance emerged, indicating autonomic flexibility relevance.

## Graphical abstract

Created in BioRender, Queirolo, L. (2026), https://BioRender.com/850077a. 
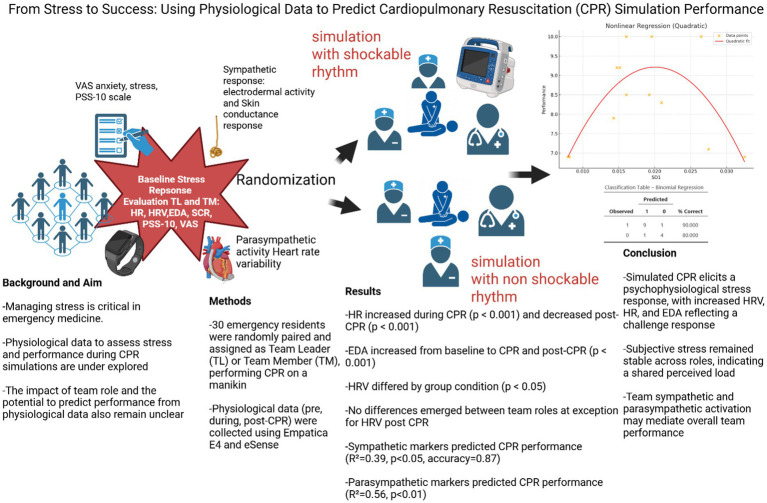

## Background: stress in emergency medicine and during cardiopulmonary resuscitation (CPR)

Emergency medicine is characterized by frequent exposure to critical situations that demand rapid and accurate decision-making, generating significant emotional and cognitive load for healthcare professionals (Boscolo et al., 2025; Janicki et al., 2020). Stress responses are mediated by prefrontal cortex functioning (Arnsten, 2009; Girotti et al., 2018; Plessow et al., 2011; Qin et al., 2009; Shields et al., 2016), and numerous reports highlight globally (Loeppke et al., 2017; Xiao et al., 2014) the high prevalence of occupational stress and burnout in emergency departments (EDs) worldwide—an issue also strongly evidenced among general medical students (Queirolo et al., 2024c; Quek et al., 2019). Among the most stressful events, cardiopulmonary resuscitation (CPR) represents one of the most demanding scenarios, requiring coordinated team effort under pressure, precise execution of resuscitation maneuvers, and effective communication management.

An effective team is essential during CPR to improve outcomes in patients experiencing cardiac arrest. Several studies debate about the optimal configuration, with some indicating that a four-member team represents an optimal configuration (Hunziker et al., 2011), others that teams from 4 up to 6 people are better (Tsai et al., 2020; Han et al., 2022).

Clear communication and effective task distribution among the roles are crucial for the success of the intervention (Nallamothu et al., 2018). Moreover, in cases of out-of-hospital cardiac arrest, teams of this size have been observed to perform more effectively in resuscitation maneuvers compared to larger or smaller teams, avoiding delays in the chain of survival (Martin-Gill et al., 2010). The adoption of structured protocols and the continuous training of team members remain key factors in optimizing emergency response and improving patient survival rates (Maconochie et al., 2020).

Studies have shown that negative emotions were inversely correlated with clinical performance (Xi et al., 2025), while acute stress can negatively impact clinical performance (LeBlanc, 2009; Russ et al., 2018), affecting both technical skills—such as the quality of chest compressions—and non-technical skills, including decision-making and leadership abilities (Hunziker et al., 2012).

To assess stress levels in healthcare providers involved in CPR, various psychometric tools have been employed, including the Spielberger State–Trait Anxiety Inventory (STAI-6) and the Perceived Stress Scale (PSS) (Şener et al., 2025), along with physiological parameters such as heart rate (HR), heart rate variability (HRV); a proxy of prefrontal cortex functions (Beauchaine and Thayer, 2015; Kim et al., 2018; Thayer et al., 2009), and electrodermal activity a proxy of the flight or fight system (EDA) (Queirolo et al., 2024a; Claverie et al., 2020).

EDA and HRV have shown interesting abilities to predict and monitor stress (Rahma et al., 2022; Dalmeida and Masala, 2021).

The literature suggests that the adoption of stress management strategies—such as post-event debriefing (Kam et al., 2022), high-fidelity simulation-based training, the use of breathing and visualization techniques (Lauria et al., 2017; Rudaz et al., 2017) and hypnosis—can improve the wellbeing (Fisch et al., 2020) of healthcare professionals.

The aim of this study is to analyze the relationship between stress and performance during CPR. To our knowledge, no previous study has simultaneously assessed psychological factors, HRV, EDA, and SCR in the context of simulated CPR, while also distinguishing between team roles. This integrative approach offers a novel lens on how physiological stress markers relate to performance under pressure that are essential to optimize the quality of care in critical settings by maximizing decision making and to reduce the risk of burnout among healthcare providers (Aronson et al., 2022; Sarmiento et al., 2024).

## Materials and methods

### Participants

Participants were recruited on a voluntary basis through email invitations. The inclusion criteria were being an emergency medicine resident attending the School of Emergency Medicine and consent to take part in the study. Participants Exclusion criteria were not having fulfilled the Advanced Cardiovascular Life Support (ACLS), psychiatric disease and cardiovascular pathologies. Psychophysiological measures and psychological ones were acquired from 30 healthy young emergency residents in training attending the School of Emergency Medicine of the University of Padua (Padua, Italy). Participants enrolled in the present study have signed informed consent prior to participation. The study is part of the approved studies on stress and performance by the Ethics Committee of the Department of General Psychology, University of Padua (n. 3274/2019).

### Intervention: randomization and simulation

Participants were randomly assigned using dedicated randomization software REDCap Randomization Module into 2-person dyads and allocated to one of two operational roles: team leader or team member. The randomization process accounted for potential confounding effects due to extreme differences in baseline stress levels.

Because baseline perceived stress was expected to meaningfully influence both dyadic functioning and the psychophysiological response to the simulated CPR scenario, the randomization process incorporated an *a priori* pairing constraint. Specifically, pairing a participant with very high baseline stress (PSS-10 ≥ 26) with another with very low stress (PSS-10 ≤ 13) would create dyads with large within-pair differences that could mask or distort the effect of the CPR on psychophysiological variables and therefore was prohibited. This constrained approach was implemented to reduce intra-dyad variance attributable to pre-existing stress rather than to the experimental scenario, thereby minimizing confounding and preserving sensitivity to detect stress or performance changes specifically induced by the CPR simulation. Within the allowed pairing solutions satisfying this constraint, assignment remained fully random.

### Procedure

The simulations were conducted using a standardized scenario of in-hospital cardiac arrest, either with a shockable or non-shockable rhythm. Each team consisted of two participants (a randomly assigned team leader and team member) and two nurses who collaborated in the study.

The two participants were responsible for recognizing the cardiac arrest, initiating and coordinating the Advanced Life Support (ALS) protocol (Soar et al., 2025), performing high-quality chest compressions, delivering defibrillation if appropriate, and directing the overall resuscitation efforts based on their assigned role.

The nurses, while not actively leading the scenario, had predefined supportive tasks: they followed the participants’ instructions, provided materials (e.g., defibrillator pads, airway equipment), simulated medication administration when prompted, and participated in CPR when asked. Their role was designed to simulate real clinical support while ensuring consistency across all scenarios, without influencing clinical decision-making.

This setup allowed the simulation to focus on the participants’ leadership, teamwork, and technical performance under realistic conditions.

The TM group (team members) was instructed to focus on technical execution and procedural adherence.

The TL group (team leader) was instructed to act as team leader, even in a single-rescuer setting, by verbalizing decisions and maintaining situational awareness.

This framing aimed to simulate two different cognitive loads and decision-making styles during CPR under stress.

### Measurements and data collection

Before and after the simulation, skin conductance, heart rate, and heart rate variability were measured using the Empatica E4 wristband (Schuurmans et al., 2020) (Empatica Inc.^®^, 1 Broadway, 14th floor, Cambridge MA 02142 United States), while with the eSense galvanometer (Mindfield® Biosystems Ltd., Hindenburgring 4 D-48599 Gronau, Germany) skin conductance response were also measured (see Section 2.8). In addition, the VAS questionnaire was administered to assess perceived stress levels within the team.

CPR performance was recorded using a high-fidelity manikin (Gaumard Hal), which automatically collects data on chest compression rate, depth, recoil. Data were downloaded and analyzed post-scenario. Empatica E4 physiological data in Table 1.

**Table 1:** Physiological values according to condition pre cardiopulmonary resuscitation (CPR), during cardiopulmonary resuscitation (CPR) = Sim, and post = post cardiopulmonary resuscitation (CPR) with IC 95%, HR = Heart rate in BPM, EDA = Electrodermal activity in μS, Heart rate variability in ms.

Statistic	HR pre	HR Sim	HR post	HRV pre	HRV Sim	HRV post	EDA pre	EDA Sim	EDA post
Mean	79.771	95.522	81.090	45.507	57.594	45.611	3.2113	3.7927	6.0953
95% CI mean lower bound	74.572	92.509	74.127	38.033	41.805	37.041	X	X	X
95% CI mean upper bound	84.971	98.535	88.053	52.982	73.383	54.181	X	X	X
Median	76.820	95.750	76.000	38.100	52.650	38.700	1.4800	1.9850	2.5700
SD	13.924	8.0693	18.648	18.894	31.750	21.665	4.8653	4.4178	8.4135
95% CI lower bound Bootstrap median	X	X	X	X	X	X	0.72	1.21	1.68
95% CI upper bound Bootstrap median	X	X	X	X	X	X	3.03	3.01	5.6

### Video analysis and evaluation

Two trained simulation instructors, blinded to group allocation, independently reviewed video recordings of each scenario to assess procedural adherence and identify any protocol deviations. Discrepancies were resolved by consensus. After the simulation, the two raters completed the Clinical Performance Tool (CPT) (Cheng et al., 2018). CPT was adapted according to the assigned clinical scenario (shockable or non-shockable cardiac arrest). Each scenario was associated with a dedicated checklist reflecting the expected sequence of actions and timing. For example, in the shockable rhythm scenario, specific actions were evaluated in Phase 1—Dysrhythmia, including rhythm recognition, prompt defibrillation, and safe shock delivery. This allowed for accurate and scenario-specific assessment of clinical performance (see Supplementary material).

### Sample size estimation

Sample size of 30 participants was determined based on both an *a priori* power analysis and empirical physiological data from a previous study involving a baseline–stress–recovery (return to baseline) paradigm (Queirolo et al., 2024b). The Wilcoxon signed-rank test was selected for the power analysis, as EDA data are typically not normally distributed, thereby violating a key assumption of the paired *t*-test. Given this, the non-parametric Wilcoxon (1945) test was deemed more appropriate. Based on the power analysis, a sample size of 30 would be sufficient to reliably detect a minimum effect size of *δ* ≥ 0.5 with statistical power greater than 0.80, using a one-sided test and allowing for a Type I error rate of *α* = 0.05.

### Psychological assessment

Anxiety levels were measured using a Visual Analog Scale (VAS). A VAS score higher than 4.6 indicates anxiety (Facco et al., 2013). Perceived stress over the past month was assessed using the PSS-10 scale, additionally also a Perceived Stress Visual Analog Scale was administered. For the PSS-10 (Cohen et al., 1983) stress levels are interpreted as follows: scores from 0 to 13 indicate low stress; scores from 14 to 25 indicate moderate stress; and scores of 26 or higher indicate high perceived stress. For the perceived stress VAS (Lesage et al., 2012), low stress is defined as a score below 2.21, average stress ranges from 3.62 to 5.32, and a score above 5.32 indicates high stress.

The Visual analogue scale questions are reported for clarity below:

“Please rate your current level of anxiety by marking a point on the line below.”
0 = no anxiety — 10 = extreme anxiety.
“Please rate your current level of stress by marking a point on the line below.”
0 = no stress — 10 = extreme stress.

### Physiological assessments

Physiological parameters were collected 5 min before the scenario, during and immediately after, using the eSense galvanometer and the Empatica E4, a wearable wristband device capable of measuring EDA and blood volume pulse, from which HR and HRV are derived (Schuurmans et al., 2020). EDA is a property of the skin that reflects variations in electrical conductivity in response to sweat gland activity (Shields et al., 1987), and is widely used as a sympathetic nervous system index, with very high stress-classification accuracy reported in controlled experimental settings (Klimek et al., 2023). The phasic sympathetic component of EDA; skin conductance response (SCR/min), was obtained using a galvanometric sensor. SCR values < 5 were interpreted as normal, 5–9 as mild arousal, and 10–16 as high sympathetic activation consistent with increasing acute stress (Boucsein, 2012; Queirolo et al., 2025).

SCR detection was carried out automatically through the following process:

*Baseline Monitoring (“Listening State”)*—The system continuously tracked skin conductance during a neutral, resting condition to establish a provisional physiological baseline.
*Onset Detection*—An SCR event was initiated when either (a) a progressive rise in conductance persisted for ≥ 2 s, or (b) an abrupt increase of ≥ 0.5 μS was recorded relative to the baseline. Detection was aborted if the signal dropped by > 0.1 μS within this interval, prompting an immediate return to baseline monitoring.
*Event Confirmation and Measurement*—Once onset criteria were met, the earliest value was labeled as the base level, and the response amplitude was then monitored until peak conductance was reached. SCRs were quantified both globally and within 1-min sliding windows to generate derived indexes including SCR/min.
*Recovery Phase*—An SCR was classified as complete when conductance declined by ≥ 50% of the peak amplitude relative to baseline, marking the return toward pre-event tonic conditions.

**Figure 1:**
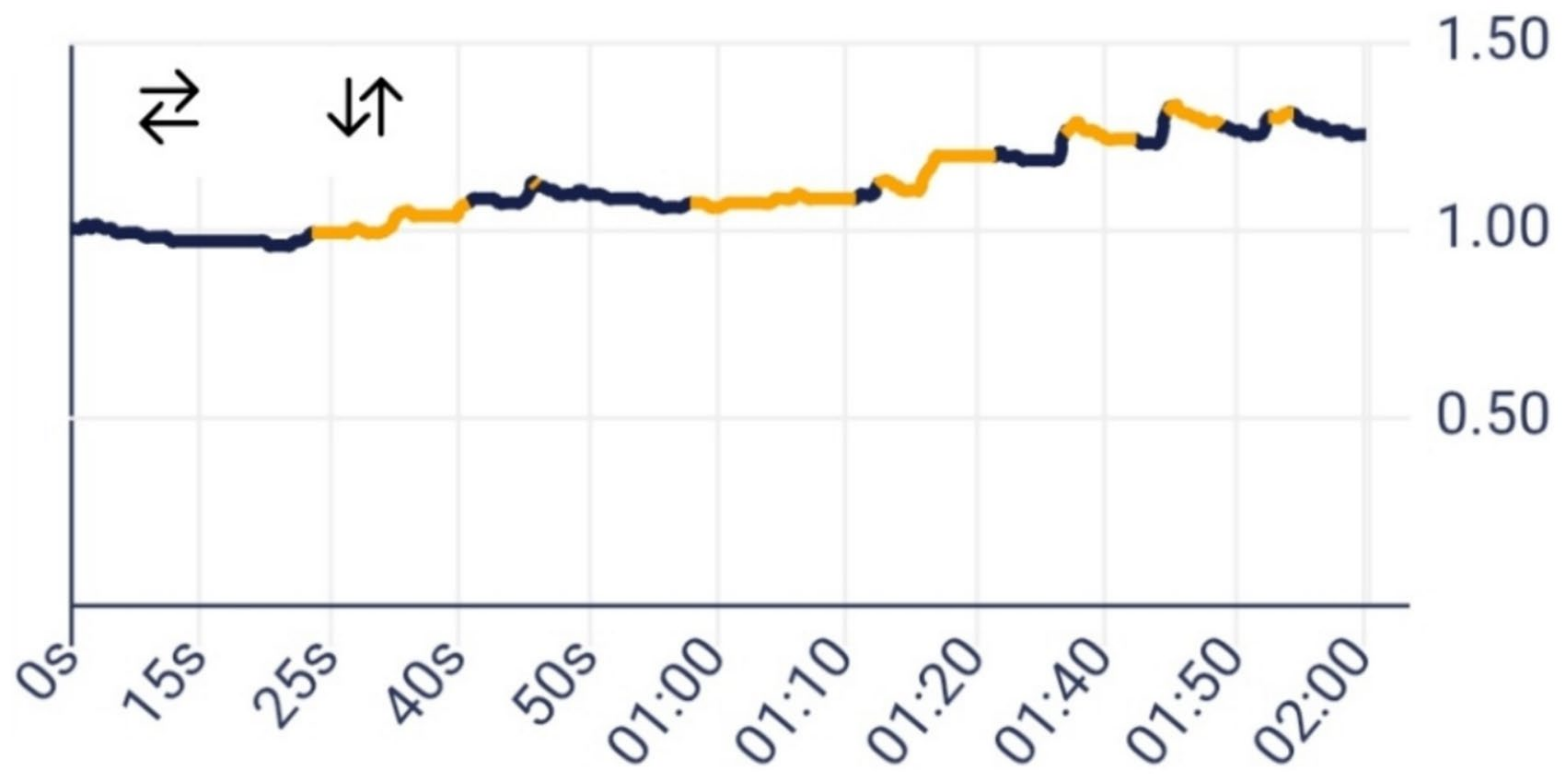
Example of electrodermal activity measured with the eSense Mindfield galvanometer app with skin conductance responses highlighted in orange. EDA expressed in μS, time in seconds.

Images of the EDA signal, from the eSense in Figure 1.

Heart rate, expressed in beats per minute (bpm), was derived from blood volume pulse using Empatica’s proprietary algorithms, which also provide inter-beat intervals (IBI) from the photoplethysmography (PPG) signal. HRV it is a parasympathetic index (Tulppo et al., 1996) associated with vagal activity and is measured using the Root Mean Square of Successive Differences (RMSSD) between inter-beat intervals from IBI. It involved the determination of the successive time differences between normal heartbeats, computing their mean, and then taking the square root of that mean to obtain the RMSSD value (Ernst, 2017). Furthermore, Standard Deviation 1; SD1—the short-term variability component derived from the Poincaré plot HRV, during first minute and half of simulation was calculated by extracting the geometrical properties of RMSSD averaged in 10 s window (to highlight differences in shorter period of time, being more robust to artifacts under stress) (Chand et al., 2024).

Although SD1 and RMSSD share a mathematical relationship at rest (approximated as SD1 = RMSSD / √2) and both index measure short-term, vagally mediated HRV, prior claims of direct equivalence (Ciccone et al., 2017) do not hold in practice during acute stress or non-stationary physiological states, including physical exertion or rapid autonomic disturbance. Consequently, the two metrics cannot be considered functionally interchangeable under CPR-relevant stress dynamics.

During CPR simulations, IBI signals become noisy and non-stationary due to motion and acute autonomic perturbation, increasing RMSSD instability when computed in raw form.

**Figure 2:**
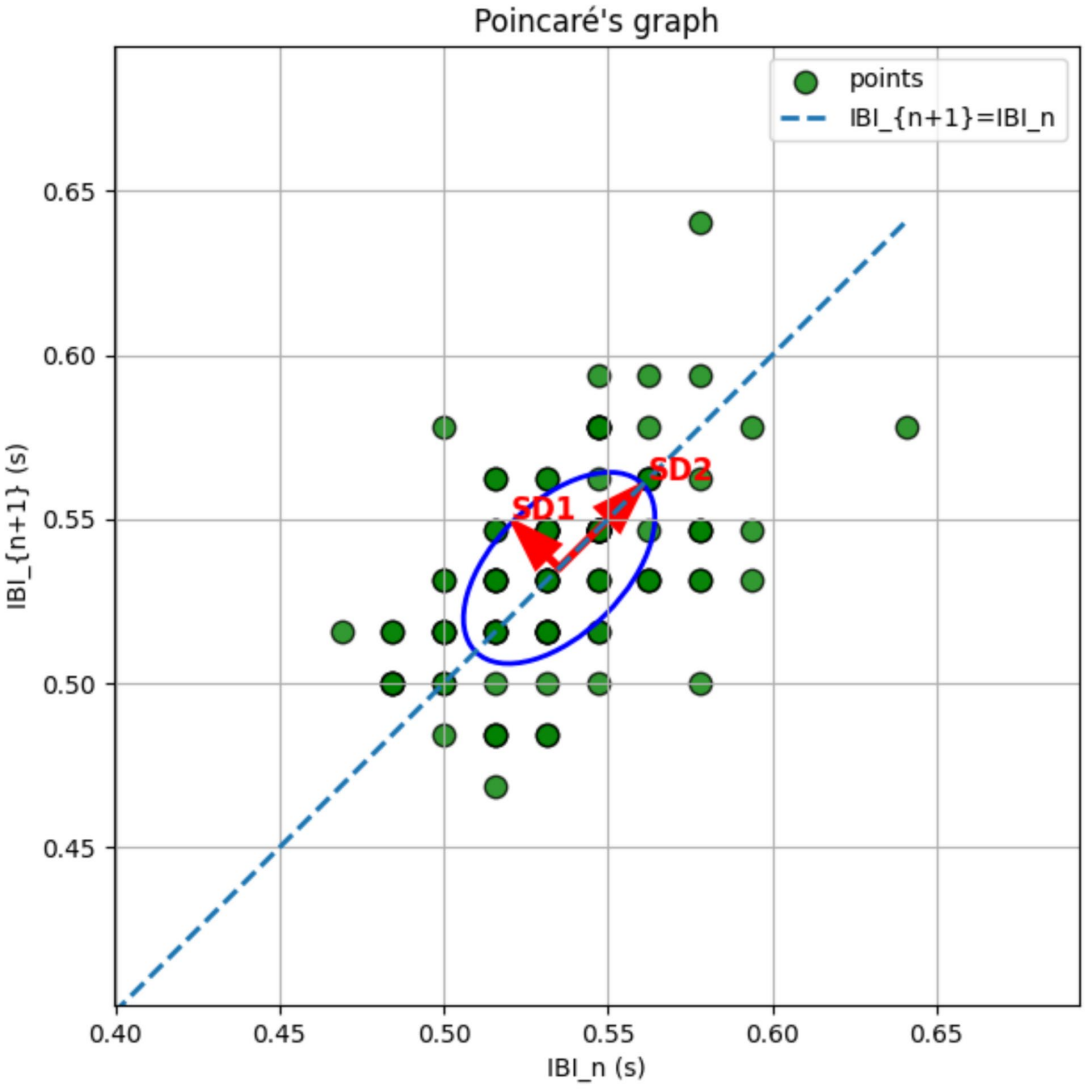
SD1 is a nonlinear heart rate variability (HRV) metric derived from Poincaré plot analysis. A Poincaré plot is a scatterplot where each IBI (RR interval) is plotted against the next one (IBIₙ vs. IBIₙ₊₁). SD1 quantifies the short-term variability of heartbeats. From a mathematical perspective it represents the semi-minor axis (the short axis) of the ellipse, while from a physiological perspective a parasympathetic (vagal) index similar, but not equal, to short-term HRV metrics such as RMSSD.

More specifically, SD1 is a short-term index of HRV derived from Poincaré plot analysis, which maps the interval between one heartbeat and the next (NN interval) against the following NN interval. Mathematically, SD1 represents the standard deviation of the dispersion of points perpendicular to the line of identity (45° diagonal), quantifying instantaneous beat-to-beat variability, for a clearer idea of SD1 (see Figure 2).

Tulppo et al. (1996) demonstrated that while SD1 and RMSSD strongly correlate at rest, this correlation significantly weakens during exercise or after parasympathetic blockade. This divergence arises because although measuring the same process RMSSD is more sensitive to noise and transient irregularities.

SD1, derived from Poincaré plot geometry, captures local dynamic patterns over slightly broader timescales, and remains more stable in dynamic or noisy environments as we expect during CPR. Thus, under stress, SD1 and RMSSD no longer represent interchangeable indices of vagal modulation, and SD1 may provide more robust data.

The blood volume pulse was measured using a PPG sensor, which can be affected by motion or pressure artifacts leading to missing data (Pollreisz and TaheriNejad, 2022). Artifacts were removed by discarding zero values and isolated outlier data points. EDA analysis included extraction of the skin conductance level, using silver-coated copper electrodes with a brass base. The threshold for significant signal amplitude was set to a minimum rise of 0.005 μSiemens. Spikes were manually removed and ±2 SD signal as well.

Physiological data were labeled according to one of three monitoring periods: Pre CPR = “pre,” CPR = “Sim” and Post CPR = “post.” Data for each subject were then aggregated by period, with mean values calculated for analysis.

Data were processed and analyzed using Jamovi, Python and Matlab.

### Statistical methods

First, the normality of the recorded variables was assessed using the Kolmogorov–Smirnov test. Variables showing a normal distribution were analyzed using Student’s *t*-tests, whereas non-normally distributed variables were analyzed using non-parametric tests (Wilcoxon signed-rank test). Parametric and non-parametric repeated-measures ANOVA models (ANOVA and Friedman’s test, respectively) were fitted depending on the distributional assumptions. Prior to model fitting, normality of residuals and homoscedasticity of variances (Bartlett’s test) were evaluated.

A repeated-measures ANOVA with three condition levels (pre, simulation, post) was conducted to determine whether the primary outcomes (dependent variables: physiological measures, including electrodermal activity [EDA], heart rate variability [HRV], and heart rate [HR]) differed significantly across conditions. To predict performance outcomes, binomial logistic regression, as well as linear and non-linear regression models, were fitted using psychophysiological data. Performance was operationalized using CPT scores, which were converted to a scale from 0 to 10. For each team member (TM)–team leader (TL) dyad, the mean physiological values were computed and used as predictors in the regression models.

## Results

The sample consisted of 30 participants with a mean age of 30 years (SD = 2.74).

Moderate levels of stress were observed across participants at baseline, as indicated by physiological (SCR = 6.193 ± 3.23) and subjective measures (VAS-Stress = 3.89 ± 1.93; VAS-Anxiety = 3.93 ± 1.98; PSS-10 = 16.67 ± 6.64). No significant differences were found in VAS stress or anxiety scores between pre- and post-CPR conditions.

Baseline physiological values were within normative ranges: HR (79.77 ± 13.92 bpm), HRV (RMSSD = 45.51 ± 18.89 ms) and EDA (Median = 1.4800 [0.72–3.03] 95% CI bound Bootstrap median).

**Figure 3:**
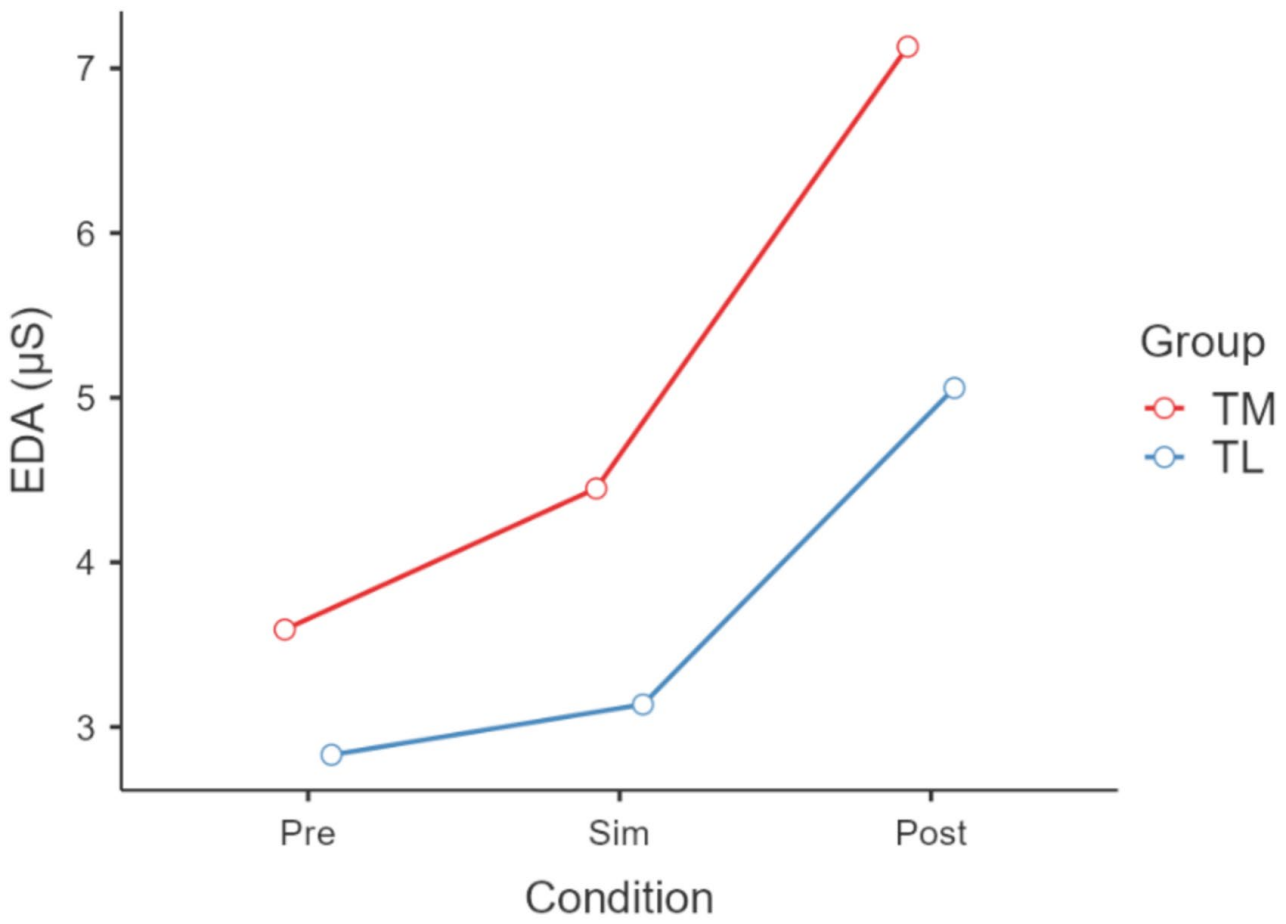
Electrodermal activity (EDA) across different conditions pre, during(Sim) and post Cardiopulmonary Resuscitation (CPR). χ^2^ = 6.2, df = 2, *p* < 0.05, with pairwise comparison showing an increase from baseline to Sim (*p* < 0.001) and an increase from baseline to post simulation (*p* < 0.001).

**Figure 4:**
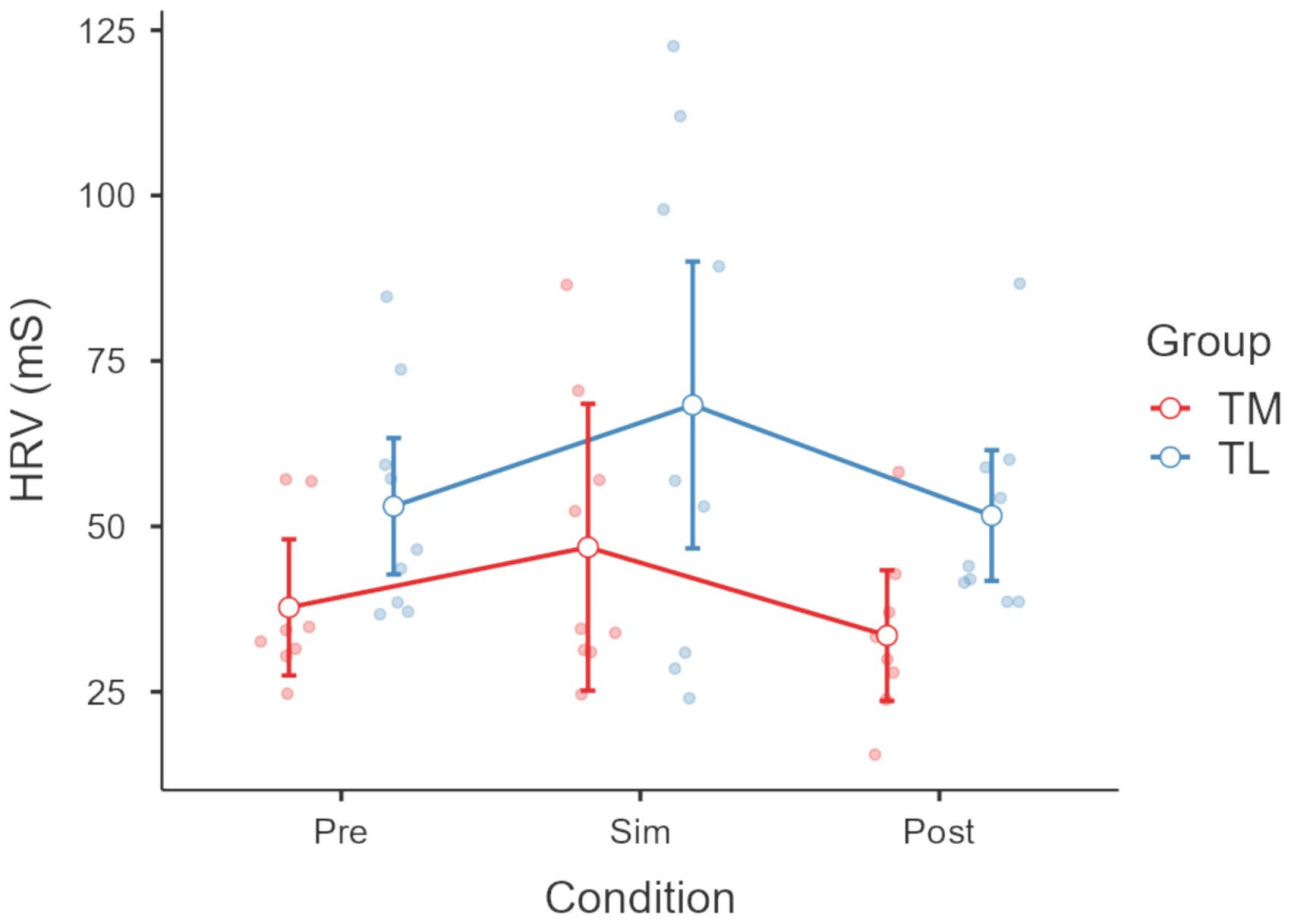
Heart rate variability (HRV) across different experimental conditions: across different conditions pre, during(Sim) and post Cardiopulmonary Resuscitation (CPR). A significant difference was also observed between team members (TM) and team leaders (TL) in the post-condition (*p* < 0.05), with higher HRV values in TL compared with TM.

**Figure 5:**
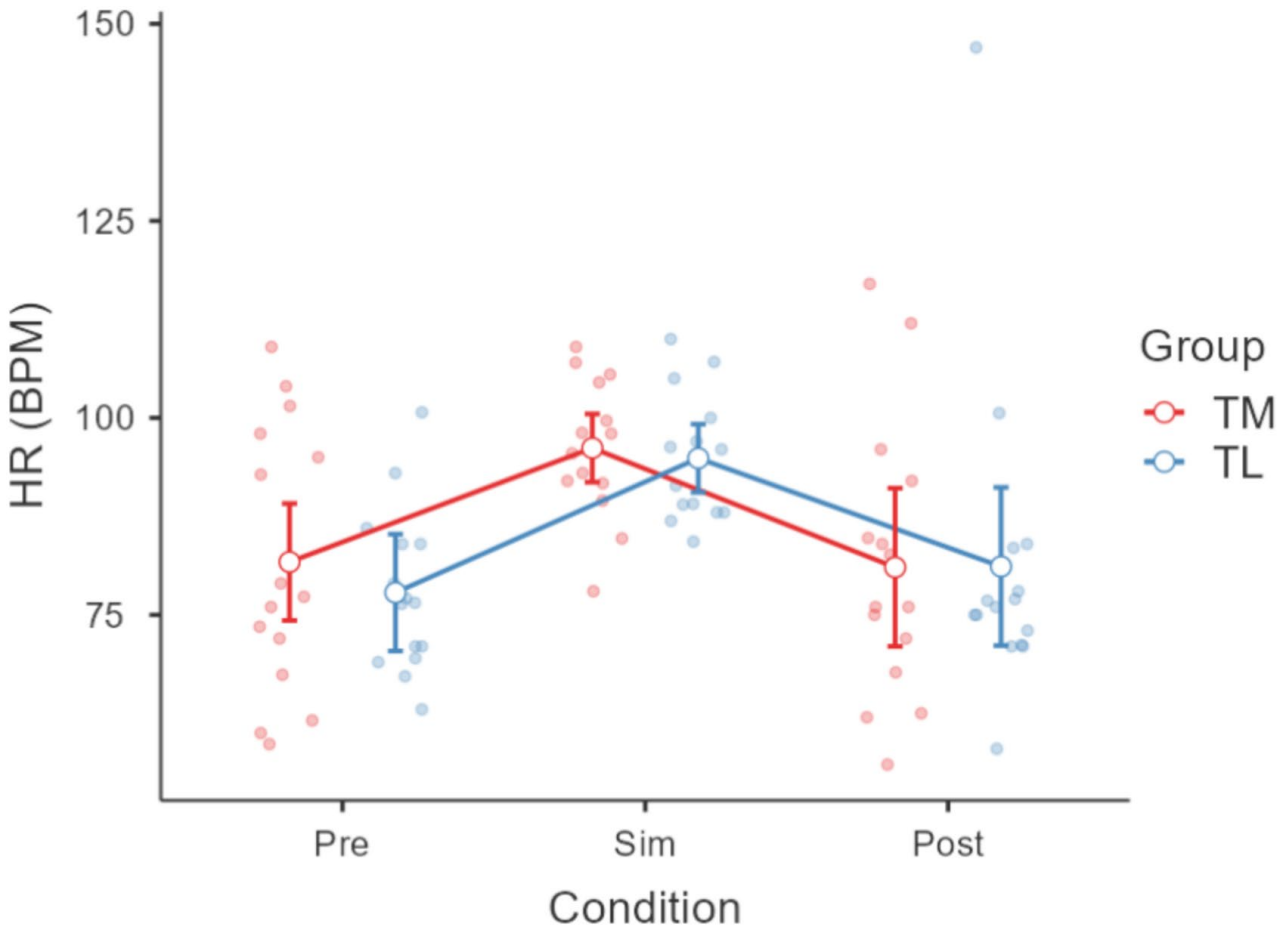
HR across different conditions pre, during(Sim) and post cardiopulmonary resuscitation (CPR). Friedman’s ANOVA showed a significant effect of condition on HR (χ^2^ = 22.30, *p* < 0.001), with *post hoc* comparisons showing HR increased during CPR (*p* < 0.001) and decreased between CPR to post-CPR (*p* < 0.001). No differences according to group membership.

Friedman’s ANOVA showed significant differences in EDA across conditions (χ^2^ = 6.20, *p* < 0.05); more specifically an increase from baseline to CPR (*p* < 0.001) and an increase from baseline to Post CPR (*p* < 0.001) (see Figure 3). ANOVA also revealed a significant effect of condition on HRV (*p* < 0.05), with TL showing higher HRV than TM post CPR (see Figure 4). Friedman’s ANOVA showed a significant effect of condition on HR (χ^2^ = 22.30, *p* < 0.001), with *post hoc* comparisons showing HR increased during CPR (*p* < 0.001) and decreased post-CPR (*p* < 0.001) (see Figure 5).

**Figure 6:**
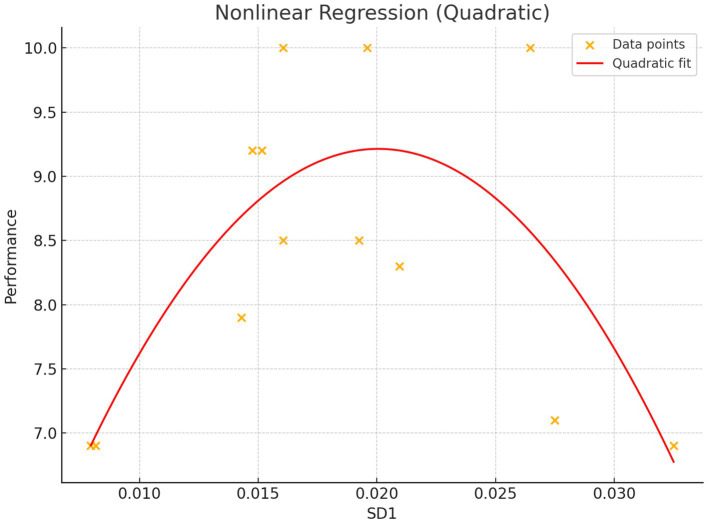
Non linear regression using SD1 to predict performance at cardiopulmonary resuscitation (CPR). “Standard Deviation 1”—the short-term variability component derived from the Poincaré plot of heart rate variability (HRV), extracted by 10 s windows in the first minute and a half and averaged based on Team Member and Team Leader values during CPR. A quadratic regression model fit to SD1 data (short-term HRV) significantly predicted CPR performance (*R*^2^ = 0.56, Coefficient *a* and *b*, *p* < 0.01). The peak of the curve occurs at SD1 = 0.0201, indicating optimal performance at intermediate levels of autonomic variability.

No significant group differences emerged between team leaders (TL) and team members (TM) in any physiological measure, with the solely exception for HRV (see Figure 4).

However, a significant direct correlation was found between TL’s pre-CPR EDA and TM’s post-CPR SCR (*R* = 0.62, df = 2, 13, *p* < 0.05).

Regression models showed that no linear regression significantly predicted CPR performance. A binomial logistic regression predicting high performance (>7.5) using sympathetic data showed a good fit (TM EDA Pre, TL EDA Pre and TL SCR pre simulation *R*^2^ McFadden = 0.39, AIC = 19.80, *p* < 0.05, accuracy = 86.7%).

More notably, a nonlinear (quadratic) regression using SD1 significantly predicted CPR performance (*R*^2^ Mc Fadden = 0.56), displaying a clear inverted U-shaped curve. Peak predicted performance (CPR = 9.17) was observed at SD1 = 0.0201. Both the quadratic and linear terms were statistically significant (*a* = −15748.2, *p* < 0.01; *b* = 631.81, *p* < 0.01; intercept = 2.88), indicating that performance decreased when SD1 deviated above or below this optimal point (see Figure 4).

## Discussion

The present study investigated psychophysiological stress responses and performance during high-fidelity CPR simulations in emergency medicine, with a focus on the role within the team (leader vs. member) and the potential for predicting performance through physiological markers.

Stress is commonly reported in emergency medicine (Janicki et al., 2020) and is often linked to decreased performance (Harvey et al., 2012), but the relationship between stress and performance is complex and not yet fully understood (Groombridge et al., 2022; Vincent et al., 2021).

Despite moderate baseline stress levels reported by participants, no significant changes were observed in subjective stress or anxiety ratings before and after the simulation. This dissociation between subjective and physiological stress is consistent with previous findings suggesting that individuals may not consciously perceive or report physiological arousal during acute stress, especially in high-demand clinical contexts (Boucher and Plusquellec, 2019; Qi et al., 2016).

Physiological data, however, revealed significant changes in HR, HRV and EDA during CPR, followed by partial recovery post-simulation (with the exception for EDA). These results confirm the validity of the emotional regulation processes described by the biopsychosocial model of arousal regulation that postulates a rise in both sympathetic and parasympathetic tone in response to challenges perceived as manageable (Blascovich and Tomaka, 1996). The activation of the autonomic nervous system in response to simulated resuscitation scenarios, was marked and supports the ecological validity of simulation-based stress induction. The persistence of elevated EDA post-simulation may indicate sustained sympathetic arousal even after the critical task has ended (claiming space for more training and experience needed to fully master the simulation with excellent emotional regulation).

Interestingly, no significant differences were observed between TL and TM in physiological stress responses, suggesting that both roles entail comparable cognitive and emotional demands within the structured simulation setting. The exception was HRV, which may function as a modulator of the broader context, potentially reflecting the pivotal role of the TL in the clinical scenario as a modulator. This finding challenges common assumptions that leadership roles inherently entail greater stress (in magnitude). Notably, differentiates the two roles the pre-simulation SCR in team leaders, which negatively correlated with CPR performance—indicating that higher fast sympathetic activation was associated with poorer outcomes, possibly reflecting a difference based on the quality of stress response rather than quantity. This pattern suggests a predominant role of rapid, phasic sympathetic reactivity in Team Leaders in shaping performance outcomes, beyond baseline tonic arousal.

On the other hand a significant association emerged between TL tonic sympathetic activation; TL EDA pre-CPR and TM post-CPR EDA, suggesting a possible mechanism of emotional transmission or synchronization within the team, supporting a complementary interpretation, whereby tonic sympathetic readiness in leaders may interact with fast phasic responses to influence subsequent team-level autonomic regulation. Overall Omnibus likelihood ratio tests indicated that Team Leader phasic sympathetic activation was the most significant individual predictor of CPR performance, whereas Team Leader and Team Member tonic sympathetic activity (EDA pre-CPR) showed marginal associations, suggesting that post-CPR TM SCR reflected the TL overall modulation.

Together, these findings indicate that TL SCR may act as a proximal physiological driver of performance, while tonic sympathetic indices contribute to a broader regulatory context, potentially mediating interpersonal synchronization within the team. This interpersonal physiological linkage may reflect the qualitative nature of stress regulation and coordination, rather than a mere difference in stress magnitude. The unique predictive value of TL SCR could thus represent the added cognitive and emotional load borne by leaders due to their need for rapid decision-making under pressure, highlighting the unique physiological cost of leadership during team-based resuscitation. The phasic sympathetic reactivity of team leaders may shape the subsequent tonic sympathetic level in team members, suggesting a form of emotional transference or contagion within the team dynamic. This unique marker may reflect the equal accountability embedded in collaborative team-based simulation and the most prominent relevance of fast action accountability for TL.

Notably, a quadratic relationship between SD1 and CPR performance was identified and its relevance in a very narrow amount of time (90 s) as previously highlighted by the scientific literature (Hunziker et al., 2011). This nonlinear model suggests that both very low and very high autonomic variability are associated with poorer performance, with peak performance observed at SD1 = 0.0201, confirming the existence of an optimal HRV window for task and adaptive regulation. This aligns with the neurovisceral integration model (Thayer and Lane, 2000), and the Yerkens and Dodson’s model (Yerkes and Dodson, 1908) (the reversed U shape between performance and anxiety here highlighted by SD1) which joint together propose that efficient physiological regulation supports cognitive flexibility and executive function—key components in managing dynamic clinical scenario as well.

The absence of predictive value in linear models further emphasizes the importance of considering nonlinear, binomial and individualized physiological patterns as already previously underlined by scientific literature, where experience is not enough (Krage et al., 2014) and subjective perception is relevant (Vincent et al., 2021); training it is necessary when dealing with performance in complex tasks. These findings underscore the potential for HRV and EDA as biomarkers of functional readiness, though further validation is needed in real-world clinical settings.

To our knowledge, this is the first study to simultaneously examine multiple physiological stress markers (HR, HRV, SD1, EDA, SCR) and psychological one, within role-differentiated CPR teams, highlighting the unique predictive contribution of parasympathetic and sympathetic reactivity in explaining CPR outcomes variability revealing their relevance as meaningful predictors of performance.

### Limitations and future directions

Several limitations should be acknowledged. The sample size, while based on power analysis, remains relatively small. Another limitation is the potential influence of wearable sensor sensitivity and signal noise, particularly under movement-related stress during CPR maneuvers Additionally, the study was conducted in a simulated environment, which, despite its realism, may not fully replicate the pressures of actual clinical emergencies. Future studies should include longitudinal designs, larger samples, and real-world data to validate the predictive utility of physiological indicators like SD1 and SCR.

## Conclusion

This study demonstrates that high-fidelity CPR simulation elicits robust psychophysiological responses that are not fully reflected in subjective stress reports, highlighting the value of physiological measures in high-demand clinical tasks. Although Team Leaders and Team Members showed comparable levels of autonomic activation, differences in autonomic regulation, rather than stress magnitude, were critical for performance. Phasic sympathetic reactivity in Team Leaders (SCR) emerged as the strongest physiological predictor of CPR outcomes, underscoring the importance of rapid autonomic dynamics during time-critical decision-making. Tonic sympathetic activity contributed to a broader regulatory context and was associated with subsequent autonomic modulation within the team. In addition, performance followed a nonlinear relationship with parasympathetic regulation, with optimal outcomes observed within a narrow SD1 window. Together, these findings suggest that effective team-based resuscitation depends on the timing and coordination of autonomic responses, providing novel insights into the physiological mechanisms underlying leadership and performance in emergency care.

## Data Availability

The raw data supporting the conclusions of this article will be made available by the authors, without undue reservation.
