# Supplementary material for: From stress to success: using physiological data to predict cardiopulmonary resuscitation simulation performance
Source: Front Psychol. 2026 Mar 5;17:1659195. doi: 10.3389/fpsyg.2026.1659195 (PMC12999406; doi:10.3389/fpsyg.2026.1659195)
Supplement: Supplementary file 1 [file Data_Sheet_1.pdf]

PHASE 1: ASYSTOLE.

Task	0 Point	1 Point	2 Points
Pulse check	• Not done	• >30 s<br>• Peripheral pulse<br>• After CPR started or epinephrine given	• <30 s and in sequence
CPR	• Not done	• Done without pulse check<br>• Done after epinephrine given<br>• >30 s after pulselessness recognized	• <30 s after pulselessness recognized and before epinephrine
ECG	• Not done	• Done without clinical assessment of circulation<br>• Done before CPR if pulselessness recognized<br>• Done after epinephrine<br>• >60 s	• Done after CPR started for pulselessness and before other therapy
IV/IO access	• Not done<br>• Only done once need for IV med recognized	• IV instead of IO<br>• >60 s	• IO in <60 s
Epinephrine	• Not done	• Called for without pulse check<br>• Called for without CPR<br>• Called for without via ETT<br>• >30 s after pulselessness recognized	• Called for after pulse check and CPR within 30 s of pulselessness recognition
Pulse recheck after ROSC	• Not done (includes ROSC never achieved)	• 30 s after ROSC<br>• Peripheral pulse check	• Central pulse checked within 30 s of ROSC
Defibrillation	• Called for	• Never called for	

CPR, cardiopulmonary resuscitation; ECG, electrocardiography; ETT, endotracheal tube; med, medicine; ROSC, return of spontaneous circulation.
Scoring instrument for asystole scenario (example, with permission from Donoghue et al[3]).
Scoring instrument for other scenarios (with permission from Donoghue et al[3]).

PHASE 1. Dysrhythmia.

Task	0 Point	1 Point	2 Points	Cannot Tell
STAGE 2. VF arrest or pulseless Vtach arrest, 8 min				
Pulse check	• Not done	• >30 s after VF occurs<br>• Peripheral pulse checked	• <30 s after VF occurs AND central pulse checked	
Rhythm identification	• Not done	• Does not verbalize rhythm but demonstrates<br>• Verbalizes incorrect rhythm	• Verbalizes correct rhythm	
Effective ventilation	• Not done	• >30 s after apnea recognized<br>• Improper ventilation rate	• <30 s after apnea recognized<br>• Proper ventilation rate and ratio (if not intubated)	
CPR	• Not done	• >30 s after pulselessness recognized<br>• Poor CPR technique (wrong hand position, improper rate, disruptions in CPR, does not check pulse with CPR)	• <30 s after pulselessness recognized AND good CPR technique AND checks pulse with CPR	
Defibrillation (first)	• Not done<br>• Attempted but electricity not delivered to patient (eg, pads not on, etc.)	• Wrong dose<br>• Wrong mode<br>• >90 s after rhythm identification	• <90 s after rhythm identification AND correct dose AND correct mode	
CPR continued	• Not done	• Delayed for >30 s<br>• Poor CPR technique (wrong hand position, improper rate, disruptions in CPR, does not check pulse with CPR)	• Initiated immediately after first shock with no delay and no pulse check, AND good CPR technique AND checks pulse with CPR	
Pulse recheck #1	• Not done	• Peripheral Pulse checked<br>• Done before CPR<br>• >30 s after 5 cycles of CPR completed	• Central pulse checked<br>• Done <30 s > after 5 cycles of CPR completed	
Defibrillation (second)	• Not done<br>• Attempted but electricity not delivered to patient (eg, pads not on, etc.)	• Wrong dose<br>• Wrong mode<br>• >120 s (or >5 cycles of CPR) after last shock	• Done 120 s or 5 cycles of CPR after last shock AND correct dose AND correct mode	
Epinephrine	• Not given	• Incorrect dose<br>• Suboptimal route (ETT)<br>• Given before second defibrillation	• IV/IO epinephrine dose given<br>• Correct dose given<br>• Given after second defibrillation	
CPR continued	• Not done	• Delayed for >30 s<br>• Poor CPR technique (wrong hand position, improper rate, disruptions in CPR, does not check pulse with CPR)	• Initiated immediately after second shock with no delay and no pulse check, AND good CPR technique AND checks pulse with CPR	
Pulse recheck #2	• Not done	• Peripheral Pulse checked<br>• Done >30 s after NSR recognized or >30 s after 5 cycles of CPR completed	• Central pulse checked<br>• Done <30 s of NSR recognition OR after 5 cycles of CPR	
Rhythm identification	• Not done	• Does not verbalize rhythm but demonstrates awareness of rhythm<br>• Verbalizes incorrect rhythm	• Verbalizes correct rhythm	

CPR, cardiopulmonary resuscitation ; IV, intravenous; IO, intraosseous; NSR, normal sinus rhythm; VF, ventricular fibrillation; Vtach, ventricular tachycardia.
